# A hybrid CNN-GNN and multitask learning pipeline for improving mild diabetic retinopathy sensitivity

**DOI:** 10.1016/j.mex.2026.104023

**Published:** 2026-06-29

**Authors:** Salsabila Amalia Harjanto, Rizka Wakhidatus Sholikah, Irzal Ahmad Sabilla, Gagatsatya Adiatmaja

**Affiliations:** Department of Information Technology, Institut Teknologi Sepuluh Nopember, Surabaya, 60111, Indonesia

**Keywords:** Diabetic retinopathy, Mild diabetic retinopathy, Convolutional neural network, Graph neural network, Multitask learning, Medical image analysis, Retinal fundus images

## Abstract

Diabetic Retinopathy (DR) is a progressive microvascular complication of diabetes that can lead to irreversible vision loss if early-stage abnormalities remain undetected. Mild DR is particularly challenging to identify due to subtle micro-lesions, severe class imbalance, and limited robustness of conventional deep learning models under domain shift. To address these challenges, this article introduces a hybrid CNN-GNN multitask pipeline designed to improve sensitivity toward Mild DR.

The proposed method integrates CNN for deep feature extraction, followed by graph-based representations that explicitly model spatial relationships between retinal regions. Both grid-based and adaptive k-nearest neighbor graph construction strategies are supported. A graph neural network performs message passing on these representations, and multitask learning is applied using two prediction heads: multiclass DR grading and binary Mild vs Non-Mild classification.

• The proposed Hybrid CNN-GNN Multitask Pipeline improves sensitivity toward Mild Diabetic Retinopathy by integrating convolutional feature extraction with graph-based spatial modelling.
• Multitask supervision enables enhanced Mild DR detection without substantial trade-off in overall classification accuracy, supporting balanced screening performance.
• Validation across in-domain and cross-domain scenarios demonstrates the method's ability to consistently prioritize Mild DR detection, with evaluation protocol provided.

## Graphical abstract

**Figure:**
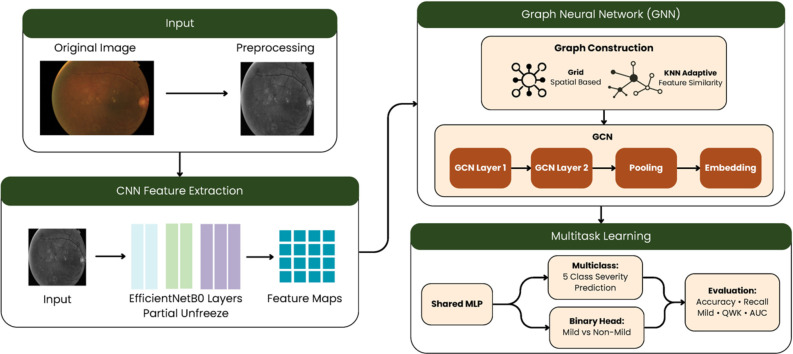

**Specifications table**

**Table:** 

**Subject area**	Computer Science
**More specific subject area**	Medical Image Analysis-Diabetic Retinopathy Screening
**Name of your method**	Hybrid CNN-GNN and Multitask Learning Pipeline for Mild Diabetic Retinopathy Detection
**Name and reference of original method**	N/A
**Resource availability**	Dataset Links - APTOS 2019: https://www.kaggle.com/c/aptos2019-blindness-detection Messidor-2: https://www.kaggle.com/datasets/mariaherrerot/messidor2preprocess IDRiD: https://www.kaggle.com/datasets/mariaherrerot/idrid-dataset DDR: https://www.kaggle.com/datasets/mariaherrerot/ddrdataset

## Background

Diabetic retinopathy (DR) is the most prevalent retinal vascular disease, resulting from the chronic effects of diabetes mellitus (DM) [1,2]. It is caused by elevated sugar levels that damage the retina and can lead to blindness if not diagnosed and treated promptly [3]. Detecting early-stage DR is challenging because retinal changes, such as microaneurysms, are extremely small, low-contrast, and often resemble normal retinal variations [4]. At this stage, patients are usually asymptomatic, leading to infrequent screenings and a lack of clinical expectation for lesions. Consequently, diagnosis heavily depends on subtle visual cues and expert judgment, which can result in higher variability and missed cases [5].

Deep learning approaches, especially CNN, have been widely adopted for automated DR detection from retinal fundus images [6,7]. However, CNN-based models often fail to capture spatial relationships between retinal lesions, leading to reduced sensitivity toward mild DR under class imbalance [8]. As a result, models with high accuracy may be inadequate for early-stage screening. Moreover, deep learning approaches reviewed by Tsiknakis et al. [9] reported that misclassification occurs more frequently for mild DR than other severity levels. The sensitivity for mild DR was extremely low at 7%. However, the evaluation found that the use of image preprocessing can increase mild DR sensitivity to 30%, but decrease the performance by 10%.

In contrast, graph-based learning has recently emerged as a complementary strategy to address these limitations. Wang et al. [10] showed that organizing CNN-extracted features into graph structures enables explicit modelling of spatial dependencies between retinal regions, improving structural representation for DR grading tasks. Furthermore, Graph Neural Networks (GNNs) can refine CNN feature embeddings and enhance robustness across datasets with varying image quality and acquisition conditions [11]. However, these approaches primarily focus on overall classification accuracy rather than sensitivity-oriented detection of minority classes.

In parallel, Multitask Learning (MTL) offers a principled solution by jointly optimizing related tasks within a shared representation space. Yu et al. [12] provided a comprehensive review of MTL, demonstrating that simultaneous optimization of related tasks improves robustness and performance under data imbalance. Moreover, Xie et al. [13] also proposed a multi-task supervised progressive learning network that jointly performs DR identification (binary) and grading (multiclass), showing improved performance compared to single-task approaches.

Despite these advances, current methods tend to explore graph-based modelling and multitask supervision separately, lacking a unified, reproducible pipeline that systematically integrates both strategies to specifically enhance Mild DR sensitivity. Moreover, most prior research evaluates performance exclusively on in-domain datasets, neglecting the vital aspect of cross-domain generalization, which is crucial for real-world application.

This work presents a hybrid CNN-GNN architecture with multitask learning specifically designed to enhance sensitivity in detecting Mild Diabetic Retinopathy while maintaining overall classification accuracy. The proposed method integrates graph-based spatial modelling with dual-task supervision to address the challenge of capturing subtle microlesions under severe class imbalance. The method is systematically validated through both in-domain evaluation and cross-domain testing on an unseen dataset.

## Method details

**Fig. 1:**
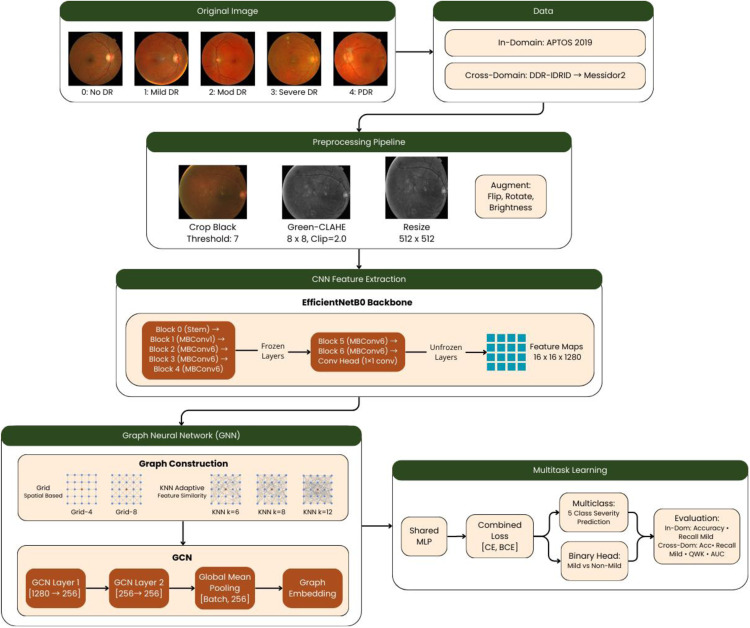
End-to-end hybrid CNN-GNN multitask pipeline for Mild DR detection.

This section describes the complete implementation of the hybrid CNN-GNN multitask pipeline, organized into eight subsections covering data handling, preprocessing, feature extraction, graph construction, neural network architectures, training strategies, multitask formulation, and validation protocols. All experiments are conducted using PyTorch 2.0.1, PyTorch Geometric 2.3.0, and timm 0.9.2 on NVIDIA A100 GPUs via Google Colab Pro. The proposed methodology is structured as an end-to-end hybrid pipeline, as shown in Fig. 1.

## Data preparation

The proposed method is evaluated under two experimental settings: in-domain and cross-domain. For the in-domain setting, the APTOS 2019 Blindness Detection dataset is used for training and validation, with Diabetic Retinopathy severity annotated into five classes: No DR (0), Mild (1), Moderate (2), Severe (3), and Proliferative DR (4). Data are partitioned using stratified 5-fold cross-validation to preserve the class distribution across folds. For cross-domain evaluation, the model is trained using a combination of DDR and IDRiD datasets and then evaluated on an unseen target dataset, Messidor-2. This dataset presents different imaging characteristics and acquisition conditions, and no domain-specific fine-tuning is applied to the target data. Identical data preparation and splitting strategies are employed across both settings to ensure consistency and to enable reliable assessment of model robustness under domain shift. Table 1 summarizes the dataset distribution for both experimental settings.

**Table 1:** Dataset distribution for in-domain and cross-domain experimental settings.

Setting	Split	Total	Class 0	Class 1	Class 2	Class 3	Class 4
**In-domain (APTOS 2019)**	Train (80%)	2929	1444	296	799	154	236
	Validation (20%)	733	361	74	200	39	59
	**Total**	**3662**	**1805**	**370**	**999**	**193**	**295**
**Cross-domain (DDR + IDRiD)**	Train (80%)	10,381	5116	522	3706	256	781
	Validation (20%)	2596	1297	130	927	64	196
	**Total**	**12,977**	**6395**	**652**	**4633**	**320**	**977**
**External test (Messidor-2)**	**Test** (n_max=100)	**100**	**100**	**27**	**100**	**75**	**35**

## Data preprocessing

All fundus images undergo a standardized preprocessing pipeline designed to remove non-informative regions and enhance subtle retinal lesions associated with early-stage Diabetic Retinopathy. The first step is automatic cropping to eliminate peripheral black backgrounds commonly present in fundus photographs [14]. This is achieved by converting the image to grayscale, applying a binary threshold at intensity level 7, and computing a tight bounding box around all pixels exceeding this threshold, ensuring that subsequent processing focuses exclusively on diagnostically relevant content.

**Fig. 2:**
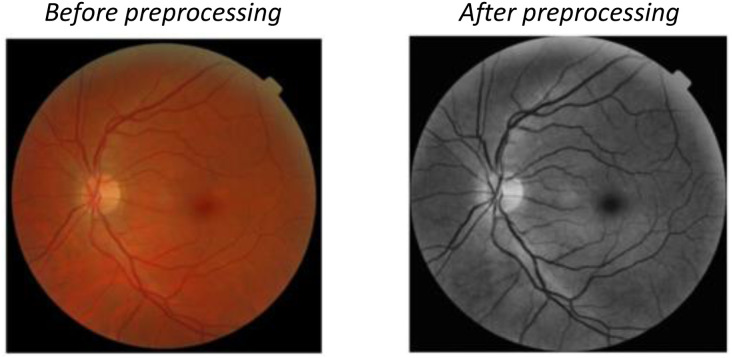
Before and after preprocessing of fundus image.

Following cropping, contrast enhancement is applied using Contrast Limited Adaptive Histogram Equalization (CLAHE) on the green channel of the RGB image. The green channel is selected due to its higher contrast sensitivity to microvascular abnormalities such as microaneurysms compared to the red and blue channels [15]. CLAHE is applied with a tile size of 8 × 8 pixels and a clip limit of 2.0 to enhance local contrast while preventing noise amplification in homogeneous regions. The enhanced green channel is replicated across three channels to maintain compatibility with the convolutional backbone. Fig. 2 shows the result of the preprocessing process.

All images are then resized to a uniform spatial resolution of 512 × 512 pixels using bilinear interpolation to match the input requirements of the CNN backbone. Lightweight data augmentation is applied during training to improve robustness against variations in orientation and illumination, including horizontal flipping (probability 0.5), small-angle rotations (range ±15°), and brightness-contrast adjustments (variation range ±0.2). These transformations are intentionally constrained to preserve the anatomical structure of the retina. Augmentation is implemented using the Albumentations library (version 1.3.1) [16].

## CNN feature extraction

Convolutional feature extraction is performed using EfficientNet-B0 as the backbone network, selected for its balanced trade-off between representational capacity and computational efficiency [17]. EfficientNet-B0 has demonstrated superior performance in medical image classification tasks, including diabetic retinopathy detection, while maintaining lower computational requirements compared to larger architectures [18]. Pre-processed fundus images are provided as RGB inputs with a fixed resolution of 512 × 512 and forwarded through the convolutional layers of EfficientNet-B0. High-level feature maps are extracted from the final convolutional block prior to global pooling and classification, preserving spatial resolution and encoding localized texture, intensity, and structural information relevant to retinal pathology.

The CNN is trained using Cross Entropy Loss and optimized with the Adam optimizer at an initial learning rate of 1 × 10⁻⁴. Training stability is maintained using a ReduceLROnPlateau scheduler with a patience of 2 epochs and a reduction factor of 0.5, while early stopping is applied if no validation improvement is observed for 5 consecutive epochs. Instead of collapsing features into a global descriptor, the resulting spatial feature maps are retained and used as node representations for subsequent graph construction, enabling localized retinal regions to be explicitly modeled in later stages.

### Graph construction

Graph construction is a central component of the proposed pipeline, enabling explicit modeling of spatial relationships between retinal regions that are not directly captured by convolutional feature extraction alone. Graphs are constructed from the spatial feature maps produced by the CNN backbone, where each spatial location corresponds to a node representing a localized retinal region.

### Node definition

Each node is defined by the feature vector extracted from a single spatial position in the CNN feature map. Given a feature map of spatial dimensions H×Wand channel depth C, the image is represented as a graph with N=H×W nodes, where each node carries a C-dimensional feature embedding. This representation preserves localized texture and intensity information while enabling relational modelling across the retina.

### Edge construction strategies

To model spatial relationships between nodes, two complementary graph construction strategies are employed: grid-based graphs and adaptive k-nearest neighbor (KNN) graphs.

In grid-based graphs, connectivity is defined directly according to the two-dimensional layout of the feature map. Two variants are considered. In the Grid-4 configuration, each node is connected to its four immediate neighbors (up, down, left, and right), while the Grid-8 configuration additionally includes diagonal neighbors, resulting in denser local connectivity. Edge relationships in grid-based graphs are implicitly governed by Euclidean distance on the feature map grid, enforcing locality and preserving anatomical continuity of retinal structures.

Adaptive KNN graphs are designed to capture more flexible, data-driven relationships between retinal regions. In this approach, each node is connected to its kmost relevant neighbors based on a combined similarity score that integrates feature similarity and spatial proximity. Feature similarity is computed using cosine similarity between CNN-derived feature vectors, while spatial proximity is computed using Euclidean distance between normalized spatial coordinates on the feature map. The two components are linearly combined using a weighting parameter α∈[0,1], defined in Eq. (1).

(1) $$\text{Similarit}y_{\text{combined}}=\alpha \cdot \text{Similarit}y_{\text{feature}}+\left(1-\alpha \right)\cdot \text{Similarit}y_{\text{spatial}}$$

**Fig. 3:**
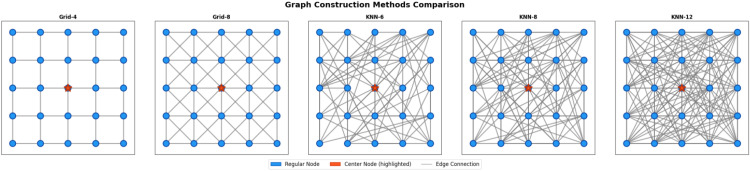
Comparison of graph construction strategies: Grid-4, Grid-8, KNN adaptive k = 6, k = 8, k = 12.

A fixed value of α=0.7is used across all experiments to ensure consistent graph topology construction. Multiple neighborhood sizes are evaluated to control graph density, including KNN-6, KNN-8, and KNN-12, enabling analysis of different local and global connectivity trade-offs. This adaptive strategy allows visually similar lesion regions to form meaningful connections even when they are not immediate spatial neighbors. The structural differences between grid-based and adaptive KNN graph construction strategies are visualized in Fig. 3, demonstrating how grid connectivity preserves strict spatial locality while KNN-based approaches enable feature-driven connections.

## GNN architecture

Graph-based learning in the proposed pipeline is implemented using a Graph Convolutional Network (GCN), which is well suited for propagating information across structured graph representations derived from retinal feature maps. The GCN operates on the constructed graphs to refine node-level representations by aggregating information from neighboring nodes through message passing [19,20].

The GNN architecture consists of two stacked graph convolution layers. The first GCN layer maps the input node features of dimension C(corresponding to the CNN feature channels) to a 256-dimensional hidden representation, followed by batch normalization and ReLU activation to stabilize training and introduce non-linearity. The second GCN layer further reduces the feature dimensionality to 128 dimensions, again followed by batch normalization and ReLU activation. This hierarchical design enables progressive refinement of node embeddings while controlling model complexity.

Message passing is performed according to the standard GCN formulation, where each node updates its representation by aggregating normalized features from its immediate neighbors as defined by the graph topology. This mechanism allows information from spatially or feature-related retinal regions to be integrated, enhancing contextual awareness for subtle lesion patterns.

After message passing, node-level embeddings are aggregated into a graph-level representation using global mean pooling, producing a fixed-length embedding vector for each retinal image regardless of graph size. This pooled embedding is then forwarded to a multilayer perceptron (MLP) classifier head, composed of a 128-to-64 fully connected layer with ReLU activation, followed by a final linear layer that maps to the target number of output classes.

**Fig. 4:**
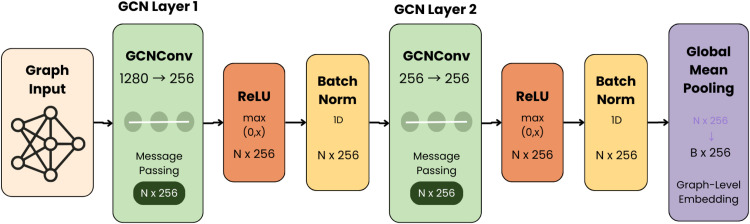
Graph Neural Network architecture with two-layer GCN and pooling.

The graph-based components are optimized using the same training configuration as the CNN backbone, including CrossEntropyLoss, Adam optimizer with a learning rate of 1 × 10⁻⁴, ReduceLROnPlateau scheduling, and early stopping. This shared configuration ensures stable joint optimization across the pipeline. The complete GNN architecture is illustrated in Fig. 4.

### Training strategy

Training of the proposed hybrid CNN-GNN pipeline is conducted in two sequential stages to ensure stable optimization and effective coupling between convolutional feature extraction and graph-based relational modelling.

In the first stage, the CNN backbone is optimized independently while all graph-based components are excluded from training. This stage allows the convolutional network to learn robust low and mid-level retinal representations without interference from graph message passing. The resulting spatial feature maps are treated as fixed inputs for subsequent graph construction.

In the second stage, joint fine-tuning is performed by unfreezing selected layers of the CNN backbone and optimizing the full CNN-GNN architecture end-to-end. Specifically, Block 5, Block 6, and the convolutional head of EfficientNet-B0 are unfrozen, while earlier layers remain frozen to preserve low-level feature representations. This partial unfreezing strategy balances adaptability to graph-based feedback with training stability. During joint fine-tuning, graph structures constructed using Grid-4, Grid-8, KNN-6, KNN-8, and KNN-12 configurations are used to propagate relational information across retinal regions.

## Multitask learning

The final stage of the proposed methodology employs a multitask learning (MTL) strategy to explicitly improve sensitivity toward Mild Diabetic Retinopathy (DR) while maintaining robust multiclass grading performance. The multitask formulation extends the CNN-GNN architecture obtained from the joint fine-tuning stage.

Multitask learning is implemented using a graph-based classifier with two task-specific output heads built on top of a shared GNN encoder. The pooled graph embedding is projected into a lower-dimensional latent space using a lightweight multilayer perceptron, reducing the feature dimensionality by half. This latent representation is shared by both tasks and subsequently branched into two independent output heads:

1. Multiclass head, which outputs logits for five DR severity classes: No DR (0), Mild (1), Moderate (2), Severe (3), and Proliferative DR (4).
2. Binary head, which outputs a single logit representing the probability of Mild DR versus Non-Mild DR.

This shared-branching design enables gradients from both tasks to jointly shape the latent representation, encouraging sensitivity to subtle pathological patterns associated with Mild DR.

### Label formulation

The multitask framework does not require additional annotations beyond the original DR severity labels. Binary labels for the Mild-versus-Non-Mild task are derived directly from the multiclass labels using a deterministic mapping: samples labelled as Mild DR are assigned a binary label of 1, while all other severity levels are assigned a label of 0. This strategy preserves label consistency across tasks and avoids reliance on auxiliary datasets.

### Loss formulation

**Fig. 5:**
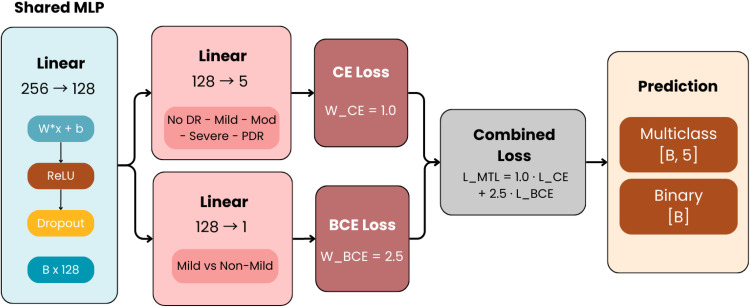
Multitask learning architecture with shared GNN encoder and dual prediction heads.

Training is supervised using a weighted combination of two loss functions corresponding to the two tasks. The multiclass loss is defined using categorical cross-entropy [21] and the binary loss is defined using weighted binary cross-entropy with logits [22] to compensate for class imbalance. The overall multitask objective is defined as a weighted sum of the two losses as shown Eq. (2).

(2) $$L_{\text{MTL}}=W_{\text{CE}}\cdot L_{\text{CE}}+W_{\text{BCE}}\cdot L_{\text{BCE}}$$

where fixed task weights $W_{\text{CE}}=1.0\;$and $W_{\text{BCE}}=2.5\;$are used throughout training to prioritize sensitivity toward Mild DR while preserving global classification performance. The multitask architecture with dual output heads is shown in Fig. 5.

### Training configuration

Multitask training is initialized from the best-performing CNN backbone obtained in the previous stage, selected based on Mild DR recall. During multitask optimization, Block 5, Block 6, and the convolutional head of EfficientNet-B0 are unfrozen, while earlier layers remain frozen to prevent catastrophic forgetting of general retinal features using the exact same configuration as CNN-GNN joint fine-tune stage. Due to the memory demands of graph-based representations, training is conducted with a batch size of 6 for a maximum of 10 epochs per fold. Graph structures and edge indices are loaded from cached files generated during earlier stages to ensure consistent graph topology across folds and training epochs. Training is performed using stratified 5-fold cross-validation, and for each fold, two model checkpoints are retained: one corresponding to the lowest validation loss and another corresponding to the highest Mild DR recall.

## Method validation

Method validation is performed by comparing the proposed hybrid CNN-GNN multitask pipeline against a CNN-only baseline using identical data splits and evaluation protocols. Method validation is performed under two evaluation settings:

1. In-domain validation, where training and testing are conducted on the same dataset distribution using stratified 5-fold cross-validation.
2. Cross-domain validation, where the model is trained on source datasets and evaluated on an unseen target dataset to assess robustness under domain shift.

All validation results are reported as averages across five folds to reduce variance caused by data partitioning and to ensure reproducibility.

## Evaluation metrics

The proposed method is validated using a set of evaluation metrics selected to reflect both sensitivity toward early-stage Diabetic Retinopathy and stability of overall classification performance.

• Mild DR recall.

Used as the primary validation metric to quantify the proportion of Mild DR cases correctly identified by the model. This metric directly reflects the method’s ability to detect early-stage disease, where false negatives are clinically critical [23].

• Global accuracy.

Reported as a secondary metric to assess overall classification stability across five DR severity levels and to ensure that improvements in Mild sensitivity are not achieved at the expense of excessive degradation in global performance [23].

• Area Under the ROC Curve (AUC).

Used in cross-domain evaluation to assess class separability under severe class imbalance. AUC provides a threshold-independent measure of model discrimination capability across datasets with heterogeneous distributions [24].

• Quadratic Weighted Kappa (QWK).

Employed to evaluate agreement between predicted and ground-truth DR severity labels in an ordinal classification setting. QWK accounts for the relative severity of misclassifications, assigning lower penalties to predictions that deviate by fewer severity levels [25].

## In-domain validation results

In-domain validation was performed on the APTOS 2019 dataset to assess the effectiveness and generalizability of the proposed framework. The experiments included three evaluation scenarios. First, we compared the proposed method with CNN-based baselines using EfficientNet-B0 trained with both standard Cross-Entropy (CE) loss and weighted Cross-Entropy (WCE) loss. This comparison aimed to evaluate how effective class weighting is in alleviating class imbalance, particularly for the minority Mild DR class. Second, we conducted an ablation study to quantify the contribution of each component within the proposed framework. This was achieved by progressively incorporating the GNN and MTL. Third, we explored the impact of different graph construction strategies by comparing the Grid method and k-Nearest Neighbours (k-NN). The Grid approach utilizes a fixed neighbourhood structure, making it well-suited for modelling uniform spatial dependencies. In contrast, the k-NN approach dynamically constructs graph edges based on feature similarity and spatial proximity, allowing for more adaptive neighbourhood representations.

All experiments were conducted using 5-fold cross-validation to evaluate the robustness of the proposed method across different data splits. Since model selection can impact the reported performance, we considered two selection criteria. The first criterion involves selecting the model with the lowest validation loss in each fold, while the second focuses on choosing the model that achieves the highest validation recall for the Mild DR class, the primary objective of this study. The experimental results are summarized in Table 2, divided into two sections corresponding to these selection criteria. For each model, the table presents the mean accuracy and Mild DR recall, along with their standard deviations across the five folds.

**Table 2:** In-domain validation results on APTOS 2019 dataset comparing CNN baseline, CNN-MTL, CNN-GNN, and CNN-GNN-MTL across different graph construction strategies for best validation loss checkpoint and best recall Mild checkpoint.

**Checkpoint by Best Validation Loss**			
**Model**	**Type**	**Mild DR Recall**	**Accuracy**
CNN baseline	Unweighted CE	0.535 ± 0.067	0.819 ± 0.008
	Weighted CE	0.654 ± 0.090	0.783 ± 0.022
CNN-MTL	Unweighted CE	0.635 ± 0.076	0.800 ± 0.018
	Weighted CE	0.654 ± 0.146	0.798 ± 0.018
CNN-GNN	Grid-4	0.622 ± 0.090	0.813 ± 0.011
	Grid-8	0.584 ± 0.107	0.817 ± 0.005
	KNN-6	0.626 ± 0.128	**0.822** **±** **0.004**
	KNN-8	0.578 ± 0.064	0.818 ± 0.014
	KNN-12	0.632 ± 0.053	0.809 ± 0.012
CNN-GNN-MTL	Grid-4	0.746 ± 0.109	0.806 ± 0.013
	Grid-8	0.727 ± 0.079	0.806 ± 0.014
	KNN-6	0.729 ± 0.074	0.795 ± 0.012
	KNN-8	0.738 ± 0.051	0.807 ± 0.017
	KNN-12	**0.765** **±** **0.077**	0.809 ± 0.006
**Checkpoint by Best Recall Mild**			
**Model**	**Type**	**Mild DR Recall**	**Accuracy**
CNN baseline	Unweighted CE	0.651 ± 0.048	**0.823** **±** **0.011**
	Weighted CE	0.722 ± 0.080	0.803 ± 0.034
CNN-MTL	Unweighted CE	0.641 ± 0.076	0.801 ± 0.022
	Weighted CE	0.665 ± 0.146	0.811 ± 0.023
CNN-GNN	Grid-4	0.672 ± 0.103	0.812 ± 0.016
	Grid-8	0.692 ± 0.039	0.812 ± 0.017
	KNN-6	0.668 ± 0.076	0.807 ± 0.015
	KNN-8	0.665 ± 0.097	0.811 ± 0.016
	KNN-12	0.678 ± 0.065	0.799 ± 0.011
CNN-GNN-MTL	Grid-4	0.795 ± 0.054	0.796 ± 0.017
	Grid-8	**0.875** **±** **0.029**	0.782 ± 0.011
	KNN-6	0.773 ± 0.087	0.799 ± 0.023
	KNN-8	0.808 ± 0.075	0.793 ± 0.029
	KNN-12	0.800 ± 0.074	0.803 ± 0.013

The results presented in Table 2 show that using WCE consistently enhances the recall of the Mild DR class within the CNN baseline, based on both model selection criteria. However, this improvement is achieved at the cost of overall classification accuracy. When the model is selected based on the lowest validation loss, WCE increases the recall for Mild DR by 0.119, but this comes with a decrease in overall accuracy of 0.036. Similarly, when using the Mild DR recall as the selection criterion, WCE improves recall by 0.071 while reducing accuracy by 0.020. These findings illustrate the effectiveness of class weighting in addressing class imbalance, although they highlight a trade-off in overall performance.

In contrast, incorporating MTL into the CNN significantly reduces the trade-off between recall and overall accuracy. While MTL preserves the recall improvements achieved by WCE, the decrease in overall accuracy becomes negligible. Additionally, when models are selected based on the highest validation recall for the Mild DR class, the CNN-MTL model not only improves Mild DR recall but also increases overall accuracy by 0.010 points compared to the CNN baseline. This suggests that MTL helps the model learn more discriminative representations that benefit both the main multiclass task and the auxiliary binary task.

The ablation study further highlights the GNN's contribution. When GNN is integrated into the CNN on its own, performance declines slightly compared to the CNN-MTL model. However, when both GNN and MTL are combined in the proposed CNN-GNN-MTL framework, the model achieves the best overall performance. Specifically, this integrated model achieves the highest Mild DR recall while maintaining competitive overall accuracy, outperforming the CNN, CNN-GNN, and CNN-MTL variants. These results indicate that the complementary strengths of GNN and MTL enable the model to better identify the minority Mild DR class without significantly compromising overall classification performance.

**Fig. 6:**
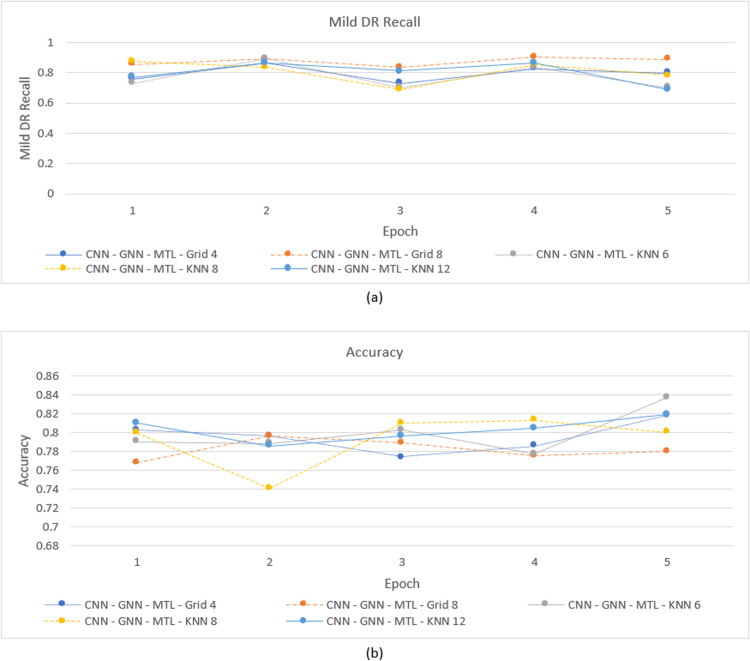
The performance of various graph construction strategies: (a) The result of Mild DR recall for each epoch; (b) The result of overall accuracy for each epoch.

Figs. 6**(a)** and **6(b)** compare the performance of the proposed CNN-GNN-MTL model using various graph construction strategies across five cross-validation folds. As seen in Fig. 6**(a)**, the Grid-8 strategy consistently demonstrates a strong and stable recall for Mild DR throughout the training process across nearly all folds. In contrast, Fig. 6**(b)** indicates that the k-NN-12 strategy provides the most stable overall accuracy, showing fewer performance fluctuations across epochs compared to the other graph construction methods. These results suggest that the choice of graph construction strategy impacts different aspects of model performance: the Grid-based graph is more effective at enhancing the recall of the minority Mild DR class, while the k-NN-based graph offers greater stability in overall classification accuracy.

The proposed MTL employs fixed task weights of $W_{\text{CE}}=1.0\;$for the multiclass classification task and $W_{\text{BCE}}=2.5\;$for the auxiliary binary classification task. These weights were selected empirically based on preliminary experiments. Several combinations of fixed task weights, together with a dynamic weighting strategy, were evaluated to investigate their influence on model performance, as summarized in Table 3. Among the evaluated configurations, the combination of $W_{\text{CE}}=1.0\;$and $W_{\text{BCE}}=2.5\;$achieved the most favourable balance between overall accuracy and Mild DR recall. A higher weight was assigned to the auxiliary binary classification loss because the primary objective of this study is to enhance the detection of the minority Mild DR class. This binary task distinguishes Mild DR from all other categories, providing a more direct supervisory for identifying features that separate Mild DR from the remaining classes. Table 3 presents the performance comparison under both model selection criteria: the lowest validation loss and the highest validation recall for Mild DR.

**Table 3:** Performance of the CNN-MTL model under different multiclass and binary loss weight configurations.

**Checkpoint by Best Validation Loss**		
**Method**	**Mild DR recall**	**Accuracy**
CNN-MTL - weighted (1.0 - 2.5)	**0.654** **±** **0.146**	0.798 ± 0.018
CNN-MTL - weighted (1.0 - 2.0)	0.586 ± 0.125	0.798 ± 0.020
CNN-MTL - weighted (1.0 - 1.5)	0.595 ± 0.121	0.794 ± 0.022
CNN-MTL - weighted (1.0 - 1.0)	0.465 ± 0.103	0.800 ± 0.021
CNN-MTL - dynamic weight	0.619 ± 0.099	**0.802** **±** **0.017**
**Checkpoint by Best Recall Mild**		
**Method**	**Mild DR recall**	**Accuracy**
CNN-MTL - weighted (1.0 - 2.5)	**0.665** **±** **0.147**	**0.811** **±** **0.023**
CNN-MTL - weighted (1.0 - 2.0)	0.593 ± 0.125	0.800 ± 0.020
CNN-MTL - weighted (1.0 - 1.5)	0.592 ± 0.123	0.798 ± 0.020
CNN-MTL - weighted (1.0 - 1.0)	0.465 ± 0.103	0.800 ± 0.021
CNN-MTL - dynamic weight	0.657 ± 0.112	0.800 ± 0.017

The final in-domain validation experiment compares the proposed CNN-GNN-MTL pipeline with several representative state-of-the-art architectures, including the baseline EfficientNet-B0, ResNet34, DenseNet121, and Vision Transformer (ViT). The comparison aims to assess the effectiveness of the proposed pipeline relative to widely used CNN- and transformer-based approaches. The results are summarized in Table 4.

**Table 4:** Performance comparison with the state-of-the-art method.

**Checkpoint by Best Validation Loss**		
**Method**	**Mild DR recall**	**Accuracy**
CNN (EfficientNet) [26]	0.654 ± 0.090	0.783 ± 0.022
CNN (ResNet34) [27]	0.738 ± 0.056	0.799 ± 0.025
CNN (DenseNet121) [28]	0.724 ± 0.089	0.793 ± 0.022
Vision Transformer [29]	**0.832** **±** **0.108**	0.786 ± 0.030
CNN - MTL - fix weighted	0.654 ± 0.146	0.798 ± 0.018
CNN - MTL - dynamic weighted	0.619 ± 0.099	0.802 ± 0.017
CNN - GNN	0.584 ± 0.107	**0.817** **±** **0.005**
CNN - GNN - MTL	0.727 ± 0.079	0.806 ± 0.014
**Checkpoint by Best Recall Mild**		
**Method**	**Mild DR recall**	**Accuracy**
CNN (EfficientNet) [26]	0.722 ± 0.080	0.803 ± 0.034
CNN (ResNet34) [27]	0.819 ± 0.055	0.718 ± 0.060
CNN (DenseNet121) [28]	0.832 ± 0.065	0.776 ± 0.038
Vision Transformer [29]	**0.902** **±** **0.028**	0.756 ± 0.038
CNN - MTL - fix weighted	0.665 ± 0.146	0.811 ± 0.023
CNN - MTL - dynamic weighted	0.657 ± 0.112	0.800 ± 0.017
CNN - GNN	0.692 ± 0.039	**0.812** **±** **0.017**
CNN - GNN - MTL	0.875 ± 0.029	0.782 ± 0.011

Among the evaluated models, the Vision Transformer achieves the highest Mild DR recall under both model selection criteria (lowest validation loss and highest validation Mild DR recall). The proposed CNN-GNN-MTL pipeline achieves a comparable Mild DR recall, with a difference of 0.027 relative to the Vision Transformer. In addition, the proposed pipeline requires substantially less training time than the Vision Transformer. Although the Vision Transformer provides the highest recall, the results indicate that the proposed pipeline offers a favourable trade-off between detection performance and computational efficiency. This balance may be particularly beneficial *in situ*ations where computational resources or model development time are limited.

An additional comparison of inference time, the number of trainable parameters, and model size is presented in Table 5 to evaluate the deployment characteristics of the proposed and baseline models. The number of trainable parameters provides an indication of model complexity, where models with a larger number of parameters generally require greater computational resources during training and inference. Similarly, model size reflects the storage requirement of the trained model, which is an important consideration for deployment, particularly in scenarios involving mobile devices, or resource-constrained environments. In addition, inference time is a practical indicator of runtime efficiency and is relevant for applications requiring timely responses, such as computer-aided clinical screening. To ensure a fair comparison, the inference time for all models was measured under the same experimental environment using Google Colab Pro equipped with an NVIDIA Tesla T4 GPU, with a batch size of one after a warm-up phase to simulate a practical deployment scenario. Table 5 shows that the proposed CNN-GNN-MTL model achieves competitive deployment characteristics by maintaining a relatively small model size while providing enhanced classification performance compared to the other evaluated methods, making it a promising candidate for practical applications in diabetic retinopathy screening.

**Table 5:** The comparison of the number of trainable parameters, model size, and inference time for the evaluated models.

**Method**	**Total Param (M)**	**Model Size (MB)**	**Inference Time (ms)**
CNN (EfficientNet) [26]	4.01	15.6	9.182 ± 1.562
CNN (ResNet34) [27]	21.29	81.3	9.027 ± 0.516
CNN (DenseNet121) [28]	6.96	27.1	17.197 ± 2.205
Vision Transformer [29]	119.11	455.4	134.883 ± 4.440
CNN - MTL	4.34	16.8	9.449 ± 1.678
CNN - GNN	4.44	17.2	16.011 ± 0.565
CNN - GNN - MTL	4.44	17.2	16.474 ± 0.651

## Cross-domain validation results

Cross-domain validation is conducted by training models on DDR- IDRiD and evaluating them on the unseen Messidor-2 test-set using best CNN-GNN-MTL model from in-domain training. This experiment assesses the robustness of the proposed method under domain shift. Table 6 summarizes the cross-domain validation performance on the Messidor-2 dataset.

**Table 6:** Cross-domain validation results on Messidor-2 test set comparing CNN baseline and CNN-GNN-MTL.

**Model**	**Mean Accuracy**	**Mean AUC**	**Mean QWK**	**Highest Mild DR Recall**
CNN baseline	0.5668 ± 0.0154	0.8753 ± 0.0117	0.6893 ± 0.0324	0.52
CNN-GNN-MTL	0.5746 ± 0.0329	0.8617 ± 0.0156	0.6623 ± 0.0611	0.76

**Fig. 7:**
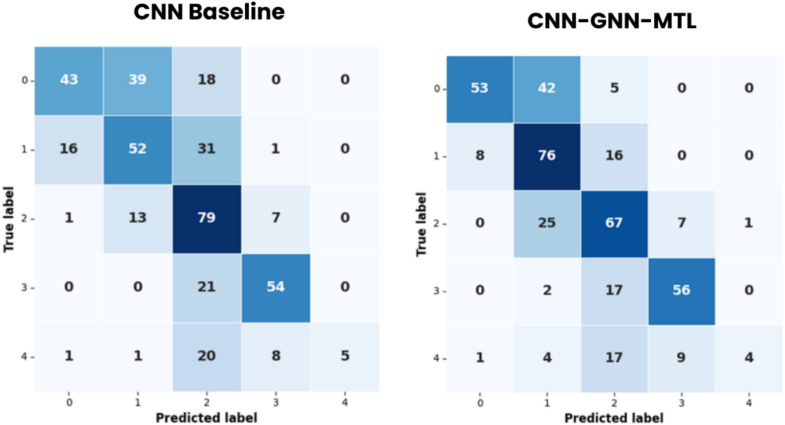
Normalized confusion matrices for cross-domain evaluation on Messidor-2: CNN baseline and CNN-GNN-MTL model showing improved Mild DR detection.

Cross-domain validation indicates an overall performance degradation when models trained on the source domain are evaluated on unseen data, which is expected under domain shift conditions. Despite this global decline, the proposed CNN-GNN-MTL pipeline consistently maintains higher performance than the CNN-only baseline. The CNN-GNN-MTL model with KNN-8 graph construction yields a 64.4% relative improvement in Mild DR recall compared to the CNN baseline. Notably, this improvement is accompanied by a slight increase in overall accuracy (+1.38%), indicating enhanced robustness. The best-performing fold reaches a Mild DR recall of 0.76, substantially exceeding the maximum baseline recall of 0.52, further supporting the effectiveness of the proposed method. Fig. 7 presents normalized confusion matrices for cross-domain evaluation, comparing the baseline and the proposed model. The CNN baseline exhibits a high rate of false negatives for Mild DR, with frequent misclassification into No DR and Moderate DR classes. In contrast, the CNN-GNN-MTL model reduces these misclassifications, indicating improved sensitivity toward Mild DR under domain shift.

## Limitations

The proposed hybrid CNN-GNN multitask pipeline demonstrates improved sensitivity toward Mild Diabetic Retinopathy detection, but several limitations should be acknowledged. First, the method relies on high-quality fundus images with sufficient resolution and contrast to construct meaningful graph representations. Images with severe degradation, excessive noise, or significant artifacts may lead to suboptimal feature extraction and graph construction, potentially reducing classification performance. Second, the graph construction process, particularly the adaptive k-nearest neighbor strategy, introduces computational overhead compared to standard CNN-only approaches. This may limit real-time deployment in resource-constrained clinical environments, although the inference time remains acceptable for batch processing applications. Third, while the method improves Mild DR recall, this improvement comes with a modest trade-off in overall accuracy, particularly when evaluated on cross-domain datasets. The degree of this trade-off may vary depending on dataset characteristics, imaging protocols, and class distribution. Fourth, the multitask learning framework requires careful tuning of task weights to balance sensitivity and specificity. The fixed weight configuration used in this study may not generalize optimally across all datasets or clinical scenarios, and domain-specific weight adjustment may be necessary. Finally, the method has been validated primarily on publicly available datasets with specific imaging conditions and annotation protocols. Performance may vary when applied to datasets acquired using different fundus camera models, lighting conditions, or field-of-view settings. Despite these limitations, the proposed method provides a systematic and reproducible framework for enhancing Mild DR detection while maintaining competitive overall classification performance.

## Ethics statements

None.

## CRediT author statement

**Salsabila Amalia Harjanto**: Conceptualization, Methodology, Validation, Formal analysis, Investigation, Data curation, Writing - Original draft, Visualization. **Rizka Wakhidatus Sholikah**: Validation, Funding acquisition, Writing - Review, Supervision, Project administration. **Irzal Ahmad Sabilla**: Supervision. **Gagatsatya Adiatmaja**: Writing - Review.

## Declaration of generative AI and AI-assisted technologies in the manuscript preparation process

During the preparation of this work, the author(s) used Grammarly and Editage-paperpal in order to improve the fluency and structure of the paragraphs. After using this tool/service, the author(s) reviewed and edited the content as needed and take(s) full responsibility for the content of the published article.

## Declaration of competing interest

The authors declare that they have no known competing financial interests or personal relationships that could have appeared to influence the work reported in this paper.

## Data Availability

The dataset used is open access and already inform inside the Resource availability table in the manuscript
